# European Headache Federation guideline on the use of monoclonal antibodies targeting the calcitonin gene related peptide pathway for migraine prevention – 2022 update

**DOI:** 10.1186/s10194-022-01431-x

**Published:** 2022-06-11

**Authors:** Simona Sacco, Faisal Mohammad Amin, Messoud Ashina, Lars Bendtsen, Christina I. Deligianni, Raquel Gil-Gouveia, Zaza Katsarava, Antoinette MaassenVanDenBrink, Paolo Martelletti, Dimos-Dimitrios Mitsikostas, Raffaele Ornello, Uwe Reuter, Margarita Sanchez-del-Rio, Alexandra J. Sinclair, Gisela Terwindt, Derya Uluduz, Jan Versijpt, Christian Lampl

**Affiliations:** 1 Department of Biotechnological and Applied Clinical Sciences – University of L’Aquila, Via Vetoio 1, L’Aquila, Italy; 2 Danish Headache Center, Department of Neurology, Rigshospitalet Glostrup, University of Copenhagen, Copenhagen, Denmark; 3 Department of Neurorehabilitation/Traumatic Brain Injury, Rigshospitalet, University of Copenhagen, Copenhagen, Denmark; 4 Hospital da Luz Headache Center, Neurology Department, Hospital da Luz Lisboa, Lisbon, Portugal; 5 Center for Interdisciplinary Research in Health, Universidade Católica Portuguesa, Lisbon, Portugal; 6 Christian Hospital Unna, Unna, Germany; 7 University of Duisburg-Essen, Duisburg, Germany; 8 Department of Internal Medicine, Erasmus MC Medical Center, Rotterdam, The Netherlands; 9 Department of Clinical and Molecular Medicine, Sapienza University, Rome, Italy; 10 1st Department of Neurology, Aeginition Hospital, School of Medicine, National and Kapodistrian University of Athens, Athens, Greece; 11 Department of Neurology, Charité Universitätsmedizin Berlin, Berlin, Germany; 12 Universitätsmedizin Greifswald, Greifswald, Germany; 13 Department of Neurology, Clinica Universidad de Navarra, Madrid, Spain; 14 Institute of Metabolism and Systems Research, College of Medical and Dental Sciences, University of Birmingham, Birmingham, B15 2TT UK; 15 Department of Neurology, University Hospitals Birmingham NHS Foundation Trust, Queen Elizabeth Hospital, Birmingham, B15 2WB UK; 16 Department of Neurology, Leiden University Medical Center, Leiden, The Netherlands; 17 Department of Neurology Istanbul Cerrahpasa Medical Faculty, Istanbul, Turkey; 18 Department of Neurology, Vrije Universiteit Brussel (VUB), Universitair, Ziekenhuis Brussel (UZ Brussel), Brussels, Belgium; 19 Department of Neurology, Headache Medical Center at the Konventhospital BHB Linz, Linz, Austria

**Keywords:** Monoclonal antibodies, Calcitonin gene-related pathway, Guideline, Migraine, Prevention

## Abstract

**Background:**

A previous European Headache Federation (EHF) guideline addressed the use of monoclonal antibodies targeting the calcitonin gene-related peptide (CGRP) pathway to prevent migraine. Since then, randomized controlled trials (RCTs) and real-world evidence have expanded the evidence and knowledge for those treatments. Therefore, the EHF panel decided to provide an updated guideline on the use of those treatments.

**Methods:**

The guideline was developed following the Grading of Recommendation, Assessment, Development, and Evaluation (GRADE) approach. The working group identified relevant questions, performed a systematic review and an analysis of the literature, assessed the quality of the available evidence, and wrote recommendations. Where the GRADE approach was not applicable, expert opinion was provided.

**Results:**

We found moderate to high quality of evidence to recommend eptinezumab, erenumab, fremanezumab, and galcanezumab in individuals with episodic and chronic migraine. For several important clinical questions, we found not enough evidence to provide evidence-based recommendations and guidance relied on experts’ opinion. Nevertheless, we provided updated suggestions regarding the long-term management of those treatments and their place with respect to the other migraine preventatives.

**Conclusion:**

Monoclonal antibodies targeting the CGRP pathway are recommended for migraine prevention as they are effective and safe also in the long-term.

**Supplementary Information:**

The online version contains supplementary material available at 10.1186/s10194-022-01431-x.

## Background

The landscape of migraine prevention has experienced relevant changes since the introduction of the monoclonal antibodies (mAbs) targeting the calcitonin gene-related (CGRP) peptide or the CGRP receptor (together referred to as CGRP-mAbs). These substances form a new class of drugs specifically developed for migraine prevention. In 2019 the European Headache Federation (EHF) issued the first guideline for the use of CGRP-mAbs for migraine prevention in adults [1]. The guideline was published to provide a first guidance on the use of CGRP-mAbs to clinicians. Since then, new drugs and randomized controlled trials (RCTs) were published together with several real-world studies. CGRP-mAbs entered the market with different prescription and reimbursement regulations for their use across countries.

Considering the new knowledge on the topic, the EHF council decided to update the 2019 guideline.

## Methods

The EHF identified a Panel of Experts consisting of the members of the working group contributing to the first guideline plus members of the EHF council; one junior member who did not participate in voting provided support for data extraction and statistical analyses. All but one member are physicians with expertise in migraine treatment; one member (AMVDB) is a pharmacologist with expertise in migraine treatment.

This guideline was organized into two parts. The first part provides evidence-based recommendations, and the second part provides Statements based on Experts Consensus.

For both parts, members of the Panel group reconsidered the clinical questions formulated in the previous guideline. Additional clinical questions were added for aspects consensually considered relevant by panel members.

### Review of the literature

The systematic review of the literature was performed according to the Preferred Reporting Items for Systematic Reviews and Meta-Analyses (PRISMA) guidelines [2, 3] from the beginning of indexing up to February 2022. We identified key papers on the use of CGRP-mAbs in individuals with migraine.

The following search string was used in both databases: “(migraine OR headache) AND (CGRP OR eptinezumab OR erenumab OR fremanezumab OR galcanezumab)”. Two investigators (SS and RO) independently screened the titles and abstracts of the publications to verify study eligibility. In the assessment of clinical questions for evidence-based reommendations, we included Phase II and Phase III primary RCTs using commercially available doses of CGRP-mAbs; we excluded reviews, other non-original articles (letters, comments, corrections to original articles), real-world studies, phase I RCTs, dose-ranging studies not using commercially available doses of CGRP-mAbs, and post-hoc and subgroup analyses of primary RCTs. For the assessment of additional questions subjected to consensus, we considered primary RCTs, their post-hoc and subgroup analyses, and real-world studies, which were selected by the Authors on the basis of clinical relevance.

Literature screening was conducted in two steps. In the first step, studies were excluded after reading the title and the abstract for clear exclusion criteria. For studies that passed the first step, the full text was assessed to decide about inclusion/exclusion. Disagreements were resolved by consensus. The reasons for exclusion were recorded and summarized. To summarize the search results, a data extraction sheet was developed including the information of interest. Papers retrieved from the literature search as well as summary tables were shared among the panelists.

### Development of evidence-based recommendations

The evidence—based recommendations were developed according to the Grading of Recommendations, Assessment, Development and Evaluation (GRADE) system [4] as the method of choice to establish recommendations.

Clinical questions were developed according to the GRADE system as Patients; Intervention; Comparison and Outcome (PICO) [4]. Outcome parameters rated as important or critical by members of the group were considered. The selected outcome parameters were reduction in monthly migraine days and proportion of individuals with migraine having at least a 50% reduction from baseline in monthly migraine days. For the studies not reporting monthly migraine days, we considered monthly headache days. For the present guideline we did not include patients-reported outcomes in the quantitative analyses. The reasons which were considered for this decision were heterogeneity of the different instruments across studies, lack of adequate information on the clinical meaningfulness of the scores for some of the instruments and the assumption that improvement in patients-reported outcomes tends to follow the improvement in monthly migraine days and responder rate.

For RCTs, the general description of the study was extracted for each publication. We extracted first author name and year of publication, full citation, study design and setting, study period, number of included individuals with migraine, diagnostic criteria for migraine, definition of migraine and headache day, migraine type, treatment type, duration of observations and treatments, and study results. Data extraction was performed by two researchers (SS and RO) and double checked by other panel members (CD, RGG, MS, JV).

For each of the selected studies one author (SS) addressed the presence of possible bias; this was checked by one panel member (JV). Thereafter, quality of evidence was addressed for selected outcomes according to the GRADE approach [4]. Information derived from RCTs was considered as high quality of evidence but the quality of single studies was downgraded in the case of study limitations such as lack of allocation concealment, lack of blinding, incomplete accounting for individuals with migraine and outcome events, selective outcome reporting bias, or other limitations such as inadequate sample or lack of sample size calculation [5]. Final quality of evidence for each of the considered outcomes was rated as high, medium, low or very low based on study design, study limitations, inconsistency, indirectness, imprecision, publication bias, effect size, dose response, and confounding factors (Table 1) [4]. Summary of findings tables were drafted using the GRADE pro statistical software considering all the outcomes considered important or critical. For the analysis of extracted data we used R, version 4.1.2 [6], and RevMan software, version 5.4. Data analysis was performed on a fixed-effects basis and results were summarized as risk ratio (RR) or risk difference (RD) and 95% confidence intervals (CI). The quality of evidence tables were prepared by a single author (SS) and then discussed and agreed in a panel meeting.

**Table 1:** Meaning of the different categories of the quality of evidence and of the strength of the recommendation according to the GRADE approach

**Grading of the quality of evidence**	
High ⨁⨁⨁⨁	We are very confident that the true effect lies close to that of the estimate of the effect
Moderate ⨁⨁⨁○	We are moderately confident in the effect estimate: the true effect is likely to be close to the estimate of the effect, but there is a possibility that it is substantially different
Low ⨁⨁○○	Our confidence in the effect estimate is limited: the true effect may be substantially different from the estimate of the effect
Very low ⨁○○○	We have very little confidence in the effect estimate: the true effect is likely to be substantially different from the estimate of effect
**Strength of the recommendation**	
Strong (↑↑)	the panel is confident that the desirable effects of adherence to a recommendation outweigh the undesirable effects
Weak (↑)	the panel concludes that the desirable effects of adherence to a recommendation probably outweigh the undesirable effects, but is not confident

### Development of the expert consensus

Questions relevant to clinical practice were drafted by experts. Questions included in the previous report were reconsidered and additional questions were added. This process was done by filling a web-based questionnaire where panel members were inquired about their opinion referring to the available clinical questions and for suggestions of new topics.

For those clinical questions the GRADE approach was not applicable, recommendations were developed as expert statements. For addressing the clinical question, information from RCTs and from observational studies was considered.

To reach a consensus on the different statements, panel discussion meetings were performed to exchange information and opinions. During the panel meeting a proposal of statements was drafted and each panel member was requested to vote on the proposed statement. Statements reaching at least a 70% agreement of the panel members were reported in the paper.

### Drafting of the statements

For evidence-based recommendations, strength (strong or weak) and direction (in favor or against) of the recommendation was determined on basis of balance between desirable and undesirable effects (Table 1) [4]. The recommendations were made exclusively based on clinical criteria. The issues of cost, reimbursement, marketing, and distribution of drugs were not considered when making the statements.

For Expert consensus statements the same wording frame of evidence-based recommendations was followed where possible. No formal rating of the quality of evidence was performed in this case.

### Final approval of the document

The guideline document underwent several rounds of revisions among the Panel members until an agreement on all the content was reached. The final version of the document was approved by all contributing authors.

## Results

This guideline is structured into two parts; the first part reports the evidence-based recommendations, and the second part reports the Expert Consensus Statements.

### Evidence-based recommendations

For the evidence-based recommendations, three PICO questions were selected. We considered phase II and phase III RCTs comparing any CGRP-mAb with placebo. Only doses finally available on the market were considered to provide evidence-based recommendations, with the only exception of fremanezumab 225 mg monthly for chronic migraine, which in RCTs had a 675 mg loading dose not used in clinical practice.

**Fig. 1:**
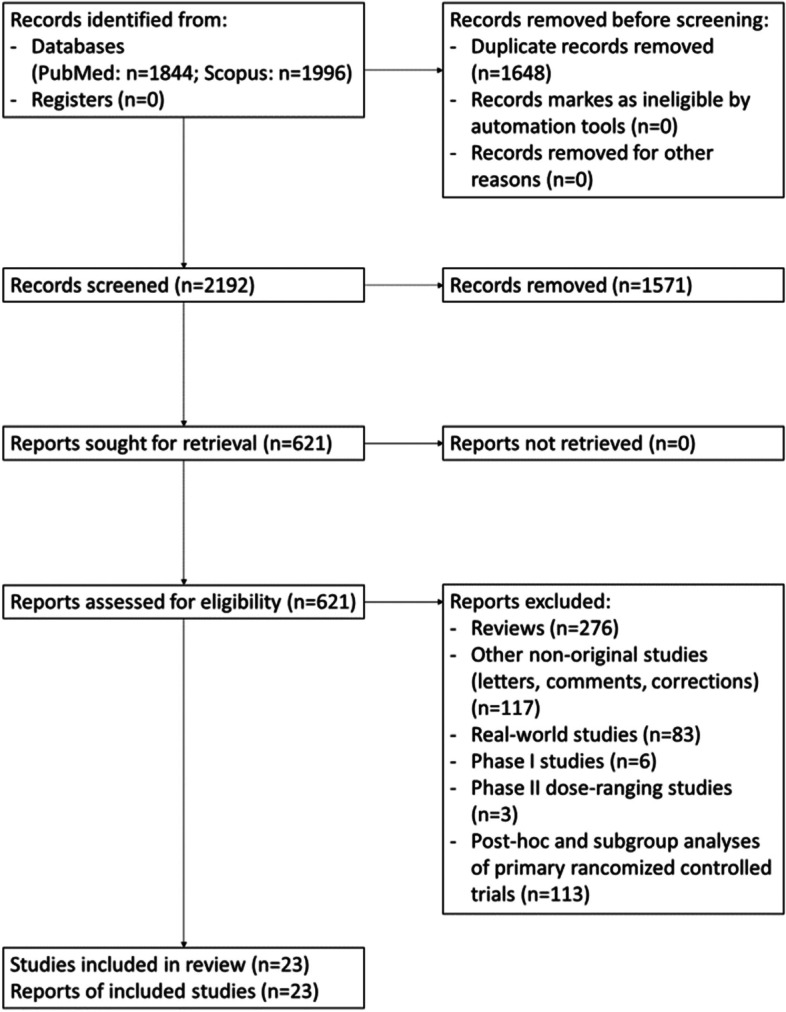
PRISMA Flowchart of study selection

**Fig. 2:**
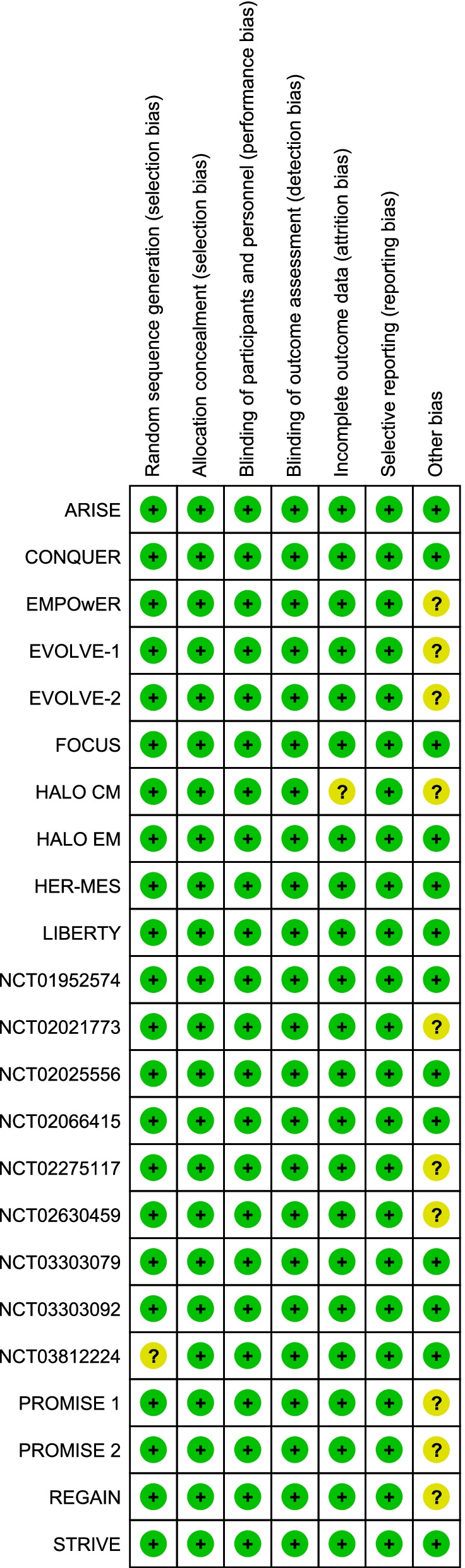
Risk of bias summary for each included study

We selected 23 studies eligible for those PICO questions [7–29]. Study selection is reported in Fig. 1, while the assessment of the risk of bias of each study is reported in Fig. 2.

The summary evidence-based recommendations are reported in Table 2.

**Table 2:** Summary of the evidence-based recommendations

Recommendation	Quality of evidence^a^	Strength of the recommendation
In individuals with episodic migraine we recommend eptinezumab, erenumab, fremanezumab and galcanezumab as preventive treatment	Eptinezumab 100 mg and 300 mg (q): moderate ⨁⨁⨁○ Erenumab 70 mg (m) and 140 mg (m): high ⨁⨁⨁⨁ Fremanezumab 225 (m) and 675 (q): high ⨁⨁⨁⨁ Galcanezumab 120 mg (m) + 240 mg (ld): high ⨁⨁⨁⨁	Strong ↑↑
In individuals with chronic migraine we recommend eptinezumab, erenumab, fremanezumab and galcanezumab as preventive treatment	Eptinezumab 100 mg and 300 mg (q): high ⨁⨁⨁⨁ Erenumab 70 mg (m): high ⨁⨁⨁⨁ Erenumab 140 mg (m): moderate ⨁⨁⨁○ Fremanezumab 225 mg (m): moderate ⨁⨁⨁○ Fremanezumab 675 mg (q): high ⨁⨁⨁⨁ Galcanezumab 120 mg (m) + 240 mg (ld): high ⨁⨁⨁⨁	Strong ↑↑
In individuals with episodic or chronic migraine we recommend erenumab over topiramate as preventive treatment because of better tolerability	Low ⨁⨁○○	Strong ↑↑

*(m)* indicates monthly, *(q)* indicates quarterly, *ld* indicates loading dose

^a^For drugs with differences in the quality of evidence across the different outcomes we provided the overall rating according to the highest quality of evidence since the risk of bias was considered minor

#### Evidence-based recommendation – question 1

**Table:** 

In individuals with episodic migraine, is preventive treatment with monoclonal antibodies targeting the CGRP pathway as compared to placebo, effective and safe?	

*Population: individuals with episodic migraine*

*Intervention: eptinezumab, erenumab, fremanezumab, galcanezumab*

*Comparison: placebo*

*Outcome: reduction in migraine days, responder rate (individuals with migraine with at least 50% reduction in migraine days), reduction in the use of acute attack medication, safety (serious adverse events or mortality)*

**Fig. 3:**
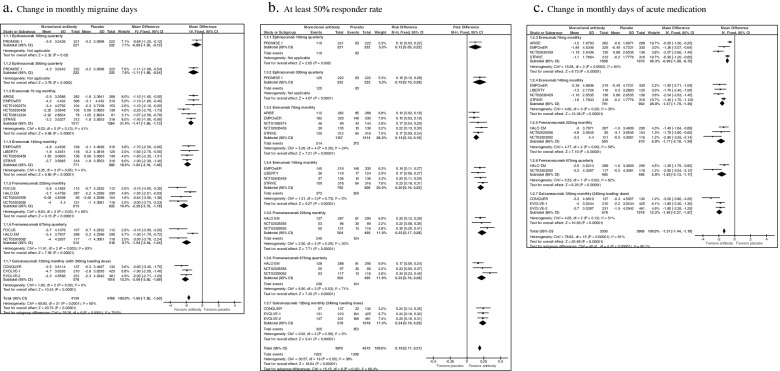
Forest plots of comparison: 1 Monoclonal antibodies vs placebo - Episodic migraine

Fifteen studies were considered for this question [7–10, 15, 16, 18, 21, 24–26, 28, 29]. The list of selected studies for question 1 is reported in Table 3. The overall results of the studies considered for question 1 are reported in Fig. 3. All the considered CGRP-mAbs (eptinezumab, erenumab, galcanezumab and fremanezumab) were associated with significant benefits considering the pre-defined outcomes as compared to placebo. No significant safety concerns were found in the different studies.

**Table 3:** Randomized placebo-controlled phase II and III clinical trials in individuals with episodic migraine

Drug/Trial	Phase	Dose	Duration	№ of participants
**Eptinezumab**				
PROMISE-1 NCT02559895 [21]	III	100 mg (q) 300 mg (q)	12 weeks	674
**Erenumab**				
NCT01952574 [16]	II	70 mg (m) 140 mg (m)	12 weeks	267
NCT02630459 [25]	II	70 mg (m) 140 mg (m)	12 weeks	475
STRIVE NCT02456740 [24]	III	70 mg (m) 140 mg (m)	24 weeks	955
ARISE NCT02483585 [7]	III	70 mg (m)	12 weeks	577
EMPOwER NCT03333109 [26]	III	70 mg (m) 140 mg (m)	12 weeks	900
NCT03812224 [29]	III	70 mg (m)	24 weeks	261
LIBERTY NCT03096834 [15]	IIIb	140 mg (m)	12-weeks	246
**Fremanezumab**				
NCT02025556 [18]	II	225 mg (m) 675 mg (m)	12 weeks	297
HALO EM NCT02629861 [12]	III	225 mg (m) 675 mg (q)	12 weeks	875
NCT03303092 [28]	III	225 mg (m) 675 mg (q)	12 weeks	357
FOCUS NCT03308968 [11]	IIIb	225 mg (m) 675 mg (q)	12 weeks	329
**Galcanezumab**^**a**^				
EVOLVE-1 NCT02614183 [10]	III	120 mg (m + 240 mg ld)	24 weeks	646
EVOLVE-2 NCT02614196 [9]	III	120 mg (m + 240 mg ld)	24 weeks	692
CONQUER NCT03559257 [8]	IIIb	120 mg (m + 240 mg ld)	12 weeks	269

Duration of all the studies is expressed in weeks and transformed as appropriate from the original study

*(m)* indicates monthly, *(q)* indicates quarterly, *ld* indicates loading dose

^a^Phase II trial NCT02163993 [30] tested a 120 mg monthly dose of galcanezumab without loading dose; therefore, it was excluded and not merged with results of other trials using a loading dose

The quality of evidence for the available compounds and for the different outcomes ranged from moderate to high (Table 4). The evidence-based recommendations for question 1 are reported in Table 2.

**Table:** 

In individuals with episodic migraine, we recommend eptinezumab, erenumab, fremanezumab and galcanezumab as preventive treatment Quality of evidence: moderate to high Strength of the recommendation: strong

**Table 4:** Summary of findings for monoclonal antibodies targeting the CGRP pathway for the prevention of episodic migraine

Outcomes	Anticipated absolute effects (95% CI)		Relative effect (95% CI)	№ of participants (studies)	Certainty of the evidence (GRADE)	Comments
	Risk with placebo	Risk with active durg				
Eptinezumab 100 mg quarterly						
Monthly migraine days	The mean monthly migraine days was – 3.2 days	Mean 0.7 days fewer (1.3 fewer to 0.1 fewer)		443 (1 RCT)	⨁⨁⨁○ Moderate^a^	Eptinezumab likely results in a reduction in monthly migraine days.
> 50% responder rate	37.4 per 100	49.8 per 100 (40.9 to 60.0)		443 (1 RCT)	⨁⨁⨁○ Moderate^a^	Eptinezumab likely results in an increase in > 50% responder rate.
Days with acute medication use	n.a	n.a	–	–	–	–
Eptinezumab 300 mg quarterly						
Monthly migraine days	The mean monthly migraine days was −3.2 days	Mean 1.1 days fewer (1.7 fewer to 0.5 fewer)		444 (1 RCT)	⨁⨁⨁○ Moderate^a^	Eptinezumab likely results in a slight reduction in monthly migraine days.
> 50% responder rate	37.4 per 100	56.3 per 100 (46.9 to 67.1)	0.19 (0.10 to 0.28)	444 (1 RCT)	⨁⨁⨁○ Moderate^a^	Eptinezumab likely results in an increase in > 50% responder rate.
Days with acute medication use	n.a	n.a	–	–	–	–
Erenumab 70 mg monthly						
Monthly migraine days	The mean monthly migraine days was −1.5 days	Mean 1.4 days fewer (1.7 fewer to 1.1 fewer)		2501 (6 RCTs)	⨁⨁⨁⨁ High	Erenumab likely results in a reduction in monthly migraine days.
> 50% responder rate	30.5 per 100	44.4 per 100 (40.7 to 48.4)	0.14 (0.10 to 0.18)	2371 (5 RCTs)	⨁⨁⨁⨁ High	Erenumab likely results in an increase in > 50% responder rate.
Days with acute medication use	The mean reduction in days with acute medication use was −0.3	Mean 0.9 fewer (1.1 fewer to 0.7 fewer)		2128 (4 RCTs)	⨁⨁⨁⨁ High	Erenumab likely results in a reduction of days with acute medication use
Erenumab 140 mg monthly						
Monthly migraine days	The mean monthly migraine days was −1.1 days	Mean 1.8 days fewer (2.2 fewer to 1.4 fewer)		1653 (4 RCTs)	⨁⨁⨁⨁ High	Erenumab likely results in a reduction in monthly migraine days.
> 50% responder rate	28.6 per 100	47.0 per 100 (42.3 to 52.0)	0.20 (0.16 to 0.25)	1698 (4 RCTs)	⨁⨁⨁⨁ High	Erenumab likely results in an increase in > 50% responder rate.
Days with acute medication use	The mean reduction in days with acute medication use was 0	Mean 1.6 fewer (1.8 fewer to 1.3 fewer)		1693 (4 RCTs)	⨁⨁⨁⨁ High	Erenumab likely results in a reduction of days with acute medication use
Fremanezumab 225 mg monthly						
Monthly migraine days	The mean monthly migraine days was −1.8 days	Mean 2.3 days fewer (2.8 fewer to 1.8 fewer)		1235 (4 RCTs)	⨁⨁⨁⨁ High	Fremanezumab likely results in a reduction in monthly migraine days.
> 50% responder rate	25.1 per 100	47.6 per 100 (41.8 to 54.0)	0.23 (0.17 to 0.28)	999 (3 RCTs)	⨁⨁⨁⨁ High	Fremanezumab likely results in an increase in > 50% responder rate.
Days with acute medication use	The mean reduction in days with acute medication use was −1.6	Mean 1.7 fewer (2.2 fewer to 1.2 fewer)		1013 (3 RCTs)	⨁⨁⨁⨁ High	Fremanezumab likely results in a reduction of days with acute medication use
Fremanezumab 675 mg quarterly						
Monthly migraine days	The mean monthly migraine days was − 1.6 days	Mean 1.9 days fewer (2.4 fewer to 1.4 fewer)		1030 (3 RCTs)	⨁⨁⨁⨁ High	Fremanezumab likely results in a reduction in monthly migraine days.
> 50% responder rate	25.1 per 100	47.0 per 100 (41.2 to 53.4)	0.22 (0.16 to 0.28)	997 (3 RCTs)	⨁⨁⨁⨁ High	Fremanezumab likely results in an increase in > 50% responder rate.
Days with acute medication use	The mean reduction in days with acute medication use was −1.4	Mean 1.6 fewer (2.1 fewer to 1.1 fewer)		811 (2 RCTs)	⨁⨁⨁○ Moderate^a^	Fremanezumab likely results in a reduction of days with acute medication use
Galcanezumab 120 mg monthly (240 mg loading dose)						
Monthly migraine days	The mean monthly migraine days was − 1.9 days	Mean 2.1 days fewer (2.5 fewer to 1.7 fewer)		1596 (3 RCTs)	⨁⨁⨁⨁ High	Galcanezumab likely results in a reduction in monthly migraine days.
> 50% responder rate	34.7 per 100	56.2 per 100 (50.3 to 62.7)	0.24 (0.19 to 0.29)	1596 (3 RCTs)	⨁⨁⨁⨁ High	Galcanezumab likely results in an increase in > 50% responder rate.
Days with acute medication use	The mean reduction in days with acute medication use was −1.7	Mean 1.9 fewer (2.3 fewer to 1.6 fewer)		1596 (3 RCTs)	⨁⨁⨁⨁ High	Galcanezumab likely results in a reduction of days with acute medication use

*CI* confidence interval, *RR* relative risk, *n.a.* not available

Explanations: ^a^Serious risk for imprecision: only 1 study, no replication

#### Evidence-based recommendation – question 2

**Table:** 

In individuals with chronic migraine, is preventive treatment with monoclonal antibodies targeting the CGRP pathway as compared to placebo, effective and safe?	

*Population: individuals with chronic migraine*

*Intervention: eptinezumab, erenumab, fremanezumab, galcanezumab*

*Comparison: placebo*

*Outcome: reduction in migraine days, responder rate (individuals with migraine with at least 50% reduction in migraine days), reduction in the use of acute attack medication, safety (serious adverse events or mortality)*

**Fig. 4:**
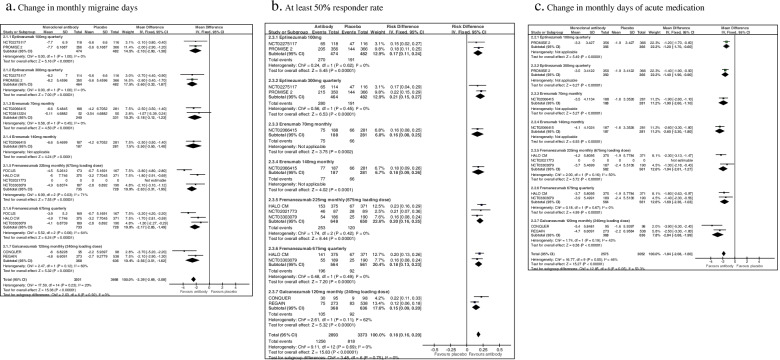
Forest plots of comparison: 2 Monoclonal antibodies vs placebo - Chronic migraine

Ten studies were considered for this question [8, 11, 13, 17, 19, 20, 22, 23, 27, 29]. The list of selected studies for question 2 is reported in Table 5. The overall results of the studies considered for question 2 are reported in Fig. 4. All the considered CGRP-mAbs (eptinezumab, erenumab, galcanezumab and fremanezumab) were associated with significant benefits considering the pre-defined outcomes as compared to placebo. No significant safety concerns were found in the different studies.

**Table 5:** Randomized placebo-controlled phase II and III clinical trials in individuals with chronic migraine

Drug/Trial	Phase	Dose	Duration	№ of participants
**Eptinezumab**				
NCT02275117 [20]	IIb	100 mg (q) 300 mg (q)	12 weeks	364
PROMISE-2 NCT02974153 [22]	III	100 mg (q) 300 mg (q)	12 weeks	1121
**Erenumab**				
NCT02066415 [19]	II	70 mg (m) 140 mg (m)	12 weeks	667
NCT03812224 [29]	III	70 mg (m)	24 weeks	261
**Fremanezumab**^**a**^				
NCT02021773 [17]	II	225 mg (m + 675 mg ld)	12 weeks	177
HALO CM NCT02621931 [13]	III	225 mg (m + 675 mg ld) 675 mg (q)	12 weeks	1130
NCT03303079 [27]	III	225 mg (m + 675 mg ld) 675 mg (q)	12 weeks	571
FOCUS NCT03308968 [11]	IIIb	225 mg (m + 675 mg ld) 675 mg (q)	12 weeks	509
**Galcanezumab**				
REGAIN NCT02614261 [23]	III	120 mg (m + 240 mg ld)	12 weeks	836
CONQUER NCT03559257 [8]	IIIb	120 mg (m + 240 mg ld)	12 weeks	193

Duration of all the studies is expressed in weeks and transformed as appropriate from the original study

*(m)* indicates monthly, *(q)* indicates quarterly, *ld* indicates loading dose

^a^The 675 mg loading dose did not enter clinical practice; however, it was tested in all trials of the 225 mg monthly dose. The difference between trials tested dose and clinical practice dosing was considered in the evaluation of quality of evidence and lead to a downgrade

The quality of evidence for the available compounds and for the different outcomes ranged from moderate to high (Table 6). The evidence-based recommendations for question 2 are reported in Table 2.

**Table:** 

In individuals with chronic migraine, we recommend eptinezumab, erenumab, fremanezumab and galcanezumab as preventive treatment Quality of evidence: moderate to high Strength of the recommendation: strong

**Table 6:** Summary of findings for monoclonal antibodies targeting the CGRP pathway for the prevention of chronic migraine

Outcomes	Anticipated absolute effects (95% CI)		Relative effect (95% CI)	№ of participants (studies)	Certainty of the evidence (GRADE)	Comments
	Risk with placebo	Risk with active durg				
Eptinezumab 100 mg quarterly						
Monthly migraine days	The mean monthly migraine days was −5.6 days	Mean 2.1 days fewer (2.9 fewer to 1.3 fewer)		956 (2 RCTs)	⨁⨁⨁⨁ High	Eptinezumab likely results in a reduction in monthly migraine days.
> 50% responder rate	39.6 per 100	57.0 per 100 (50.4 to 64.2)	0.17 (0.11 to 0.24)	956 (2 RCTs)	⨁⨁⨁⨁ High	Eptinezumab likely results in an increase in > 50% responder rate.
Days with acute medication use	The mean reduction in days with acute medication use was −1.9	Mean 1.2 days fewer (1.7 fewer to 0.6 fewer)		722 (1 RCT)	⨁⨁⨁○ Moderate^a^	Eptinezumab likely results in a reduction of days with acute medication use
Eptinezumab 300 mg quarterly						
Monthly migraine days	The mean monthly migraine days was – 5.6 days	Mean 2.6 days fewer (3.3 fewer to 1.9 fewer)		946 (2 RCTs)	⨁⨁⨁⨁ High	Eptinezumab likely results in a slight reduction in monthly migraine days.
> 50% responder rate	39.6 per 100	60.3 per 100 (53.5 to 67.8)	0.21 (0.15 to 0.27)	946 (2 RCTs)	⨁⨁⨁⨁ High	Eptinezumab likely results in an increase in > 50% responder rate.
Days with acute medication use	The mean reduction in days with acute medication use was −1.9	Mean 1.4 days fewer (1.9 fewer to 0.9 fewer)		716 (1 RCT)	⨁⨁⨁○ Moderate^a^	Eptinezumab likely results in a reduction of days with acute medication use
Erenumab 70 mg monthly						
Monthly migraine days	The mean monthly migraine days was −4.0 days	Mean 2.2 days fewer (3.1 fewer to 1.2 fewer)		571 (2 RCTs)	⨁⨁⨁⨁ High	Erenumab likely results in a reduction in monthly migraine days.
> 50% responder rate	23.0 per 100	40.0 per 100 (31.4 to 50.0)	0.16 (0.08 to 0.25)	469 (1 RCT)	⨁⨁⨁○ Moderate^a^	Erenumab likely results in an increase in > 50% responder rate.
Days with acute medication use	The mean reduction in days with acute medication use was −1.6	Mean 1.9 fewer (2.6 fewer to 1.1 fewer)		469 (1 RCT)	⨁⨁⨁○ Moderate^a^	Erenumab likely results in a reduction of days with acute medication use
Erenumab 140 mg monthly						
Monthly migraine days	The mean monthly migraine days was −4.2 days	Mean 2.5 days fewer (3.5 fewer to 1.4 fewer)		468 (1 RCT)	⨁⨁⨁○ Moderate^a^	Erenumab likely results in a reduction in monthly migraine days.
> 50% responder rate	23.0 per 100	41.0 per 100 (32.5 to 51.5)	0.18 (0.09 to 0.26)	468 (1 RCT)	⨁⨁⨁○ Moderate^a^	Erenumab likely results in an increase in > 50% responder rate.
Days with acute medication use	The mean reduction in days with acute medication use was −1.6	Mean 2.6 fewer (3.3 fewer to 1.8 fewer)		468 (1 RCT)	⨁⨁⨁○ Moderate^a^	Erenumab likely results in a reduction of days with acute medication use
Fremanezumab 225 mg monthly						
Monthly migraine days	The mean monthly migraine days was −2.2 days	Mean 2.6 days fewer (3.3 fewer to 2.0 fewer)		1463 (4 RCTs)	⨁⨁⨁○ Moderate^b^	Fremanezumab likely results in a reduction in monthly migraine days.
> 50% responder rate	18.4 per 100	39.0 per 100 (34.4 to 44.2)	0.20 (0.16 to 0.25)	1298 (3 RCTs)	⨁⨁⨁○ Moderate^b^	Fremanezumab likely results in an increase in > 50% responder rate.
Days with acute medication use	The mean reduction in days with acute medication use was −2.1	Mean 1.9 fewer (2.6 fewer to 1.3 fewer)		1123 (2 RCT)	⨁⨁○○ Low^a,b^	Fremanezumab likely results in a reduction of days with acute medication use
Fremanezumab 675 mg quarterly						
Monthly migraine days	The mean monthly migraine days was −2.2 days	Mean 2.2 days fewer (2.9 fewer to 1.5 fewer)		1461 (3 RCTs)	⨁⨁⨁⨁ High	Fremanezumab likely results in a reduction in monthly migraine days.
> 50% responder rate	16.4 per 100	34.8 per 100 (30.1 to 40.0)	0.18 (0.13 to 0.23)	1125 (2 RCTs)	⨁⨁⨁○ Moderate^a^	Fremanezumab likely results in an increase in > 50% responder rate.
Days with acute medication use	The mean reduction in days with acute medication use was −2.1	Mean 1.7 fewer (2.4 fewer to 1.0 fewer)		1125 (2 RCT)	⨁⨁⨁○ Moderate^a^	Fremanezumab likely results in a reduction of days with acute medication use
Galcanezumab 120 mg monthly (240 mg loading dose)						
Monthly migraine days	The mean monthly migraine days was −2.5 days	Mean 2.6 days fewer (3.5 fewer to 1.6 fewer)		1004 (2 RCTs)	⨁⨁⨁⨁ High	Galcanezumab likely results in a reduction in monthly migraine days.
> 50% responder rate	14.4 per 100	28.5 per 100 (23.3 to 34.5)	0.15 (0.09 to 0.20)	1004 (2 RCTs)	⨁⨁⨁⨁ High	Galcanezumab likely results in an increase in > 50% responder rate.
Days with acute medication use	The mean reduction in days with acute medication use was −2.1	Mean 2.8 fewer (3.7 fewer to 2.0 fewer)		1004 (2 RCTs)	⨁⨁⨁⨁ High	Galcanezumab likely results in a reduction of days with acute medication use

*CI* confidence interval, *RR* relative risk, *n.a.* not available

Explanations: ^a^Serious risk for imprecision: only 1 study, no replication; ^b^Serious risk for indirectness: 675 mg loading dose in RCTs

#### Evidence-based recommendation – question 3

**Table:** 

In individuals with migraine, is preventive treatment with monoclonal antibodies targeting the CGRP pathway, as compared to another migraine preventive treatment, more effective and/or tolerable?	

*Population: individuals with migraine*

*Intervention: eptinezumab, erenumab, fremanezumab, galcanezumab*

*Comparison: antiepileptics (topiramate, valproate), antidepressants (amitriptyline), beta-blockers (atenolol, metoprolol, propranolol, timolol), calcium-channel blockers (flunarizine), onabotulinumtoxinA, renin-angiotensin system inhibitors (candesartan, lisinopril)*

*Outcome: reduction in migraine days, responder ratio (individuals with migraine with at least 50% reduction in migraine days), reduction in the use of acute attack medication, discontinuation, due to adverse events, safety (serious adverse events or mortality)*

We found only one RCT which compared a CGRP-mAb versus another migraine preventive agent [14] (Table 7). In this trial erenumab (70 to 140 mg/month) was compared with topiramate (50 to 100 mg/day). The primary endpoint was medication-discontinuation due to an adverse event. The predefined secondary endpoint was set to the 50% responder rate. This study was performed in Germany only. Summary of findings for this study are reported in Table 8. Based on an intention-to-treat analysis, over the 24-week study period, there was a higher reduction in monthly migraine days with erenumab (− 5.86, SE 0.24) than with topiramate (− 4.02, SE 0.24; *p* < 0.001). More individuals with migraine achieved a > 50% reduction in monthly migraine days with erenumab than with topiramate (55.4% vs. 31.2%; odds ratio 2.76; 95% confidence interval 2.06–3.71; *p* < 0.001). In the erenumab group, 10.6% discontinued medication due to adverse events compared to 38.9% in the topiramate group (odds ratio, 0.19; 95% confidence interval 0.13–0.27; *p* < 0.001). No relevant safety concerns were observed for erenumab. The evidence-based recommendation for question 3 is reported in Table 2.

**Table:** 

In individuals with episodic or chronic migraine we recommend erenumab over topiramate as preventive treatment Quality of evidence: low Strength of the recommendation: strong

**Table 7:** Randomized controlled clinical trials in individuals with migraine comparing a monoclonal antibody targeting the CGRP pathway with another migraine preventive agent

Trial	Phase	Monoclonal antiboy/dose	Comparator/dose	Duration	№ of participants
HER-MES [14]	III	Erenumab 70-140 mg (m)	Topiramate 50-100 mg (d)	12 weeks	777

*(m)* indicates monthly, *(d)* indicates daily

**Table 8:** Summary of findings for erenumab versus topiramate for migraine prevention

Outcomes	Anticipated absolute effects (95% CI)		Relative effect (95% CI)	№ of participants (studies)	Certainty of the evidence (GRADE)	Comments
	Risk with Topiramate	Risk with Erenumab				
Monthly migraine days	The mean monthly migraine days was −4.02 days	Mean 1.84 days fewer (2.43 fewer to 1.25 fewer)	–	776 (1 RCT)	⨁⨁○○ Low^a,b^	Erenumab likely results in a slight reduction in monthly migraine days.
> 50% reduction in migraine days per month	31 per 100	56 per 100 (48 to 63)	RR 1.78 (1.50 to 2.11)	776 (1 RCT)	⨁⨁○○ Low^a,b^	Erenumab likely results in an increase in > 50% reduction in migraine days per month.
Medication discontinuation	39 per 100	11 per 100 (8 to 15)	RR 0.27 (0.20 to 0.37)	776 (1 RCT)	⨁⨁⨁○ Moderate^a^	Erenumab likely results in a reduction in medication discontinuation.

*CI* confidence interval, *RR* relative risk

Explanations: ^a^Only 1 study performed in a single country, no replication; ^b^not the primary outcome of the study

### Expert consensus statements

The summary of statements is reported in Table 9.

**Table 9:** Summary of the expert consensus statements

Question	Statement
1. When should treatment with monoclonal antibodies targeting the CGRP pathway be offered to individuals with migraine?	In individuals with migraine who require preventive treatment, we suggest monoclonal antibodies targeting the CGRP pathway to be included as a first line treatment option.
2. How should other preventive treatments be managed when using monoclonal antibodies targeting the CGRP pathway in individuals with migraine?	In individuals with episodic or chronic migraine there is insufficient evidence to make suggestions regarding the combination of monoclonal antibodies targeting the CGRP with other preventatives to improve migraine clinical outcomes
3. When should treatment efficacy in individuals with migraine on treatment with anti-CGRP monoclonal antibodies be firstly evaluated?	In individuals with episodic or chronic migraine who start a new treatment with one monoclonal antibody targeting the CGRP pathway we suggest evaluating efficacy after a minimum of 3 consecutive months on treatment
4. When should treatment with anti-CGRP monoclonal antibodies be paused in individuals with migraine?	In individuals with episodic or chronic migraine we suggest considering a pause in the treatment with monoclonal antibodies targeting the CGRP pathway after 12-18 months of continuous treatment. If deemed necessary, treatment should be continued as long as needed. In individuals with migraine who pause treatment, we suggest restarting the treatment if migraine worsens after treatment withdrawal.
5. Should individuals with migraine and medication overuse offered treatment with monoclonal antibodies targeting the CGRP pathway?	In individuals with migraine and medication overuse, we suggest offering monoclonal antibodies targeting the CGRP pathway.
6. In individuals with migraine who are non-responders to one monoclonal antibody targeting the CGRP pathway, is switching to a different antibody an option?	In individuals with migraine with inadequate response to one monoclonal antibody targeting the CGRP pathway, there is insufficient evidence on the potential benefits of antibody switch but switching may be an option.
7. In which individuals with migraine is caution suggested when considering treatment with monoclonal antibodies targeting the CGRP pathway?	We suggest avoiding monoclonal antibodies targeting the CGRP pathway in pregnant or nursing women. We suggest caution and decision on a case-by-case basis in the presence of vascular disease or risk factors and Raynaud phenomenon. We suggest caution in erenumab use in individuals with migraine with history of severe constipation.

#### Expert consensus statement – question 1

**Table:** 

When should treatment with monoclonal antibodies targeting the CGRP pathway be offered to individuals with migraine?	

##### Clinical guidance

The previous EHF guideline recommended CGRP-mAbs as a third line treatment for migraine prevention in individuals with migraine and inadequate response, lack of tolerability or lack of compliance to at least two categories of migraine preventatives [1]. Of note, in phase II and phase III trials on CGRP-mAbs, 46.3% of individuals with migraine were treatment naive or without a previous history of drug failure [7–10, 16, 17, 19, 20, 24, 26].

After the publication of the previous guideline, CGRP-mAbs became available in Europe and real-world observational studies confirmed the effectiveness of those drugs outside RCTs [31–34]. Tolerability and safety profiles were confirmed to be excellent and the adherence to treatment was not reported as a critical issue as it was with oral treatments [35–37].

CGRP-mAbs have an efficacy which is at least comparable to the efficacy of the formerly used preventive drugs. Among the oral prophylactics, high dropout rates were reported especially for amitriptyline, valproate or topiramate [37]. The major added value of CGRP-mAbs, compared to the classical preventatives, seems to be their unprecedented favorable adverse effect profile that is also associated with ease of use and high efficacy. These characteristics lead most individuals with migraine to express a clear preference for CGRP-mAbs as a first-line option [38]. Poor response in individuals with migraine may also be attributed to lack of compliance to available medical treatments because of the need of taking multiple doses of the drugs or adverse events. Additionally, CGRP-mAbs may represent a suitable option for individuals with migraine who have contraindications to other preventive treatments or in whom adverse events may be particularly challenging. Considering the overall evidence of benefits regarding the CGRP-mAbs, their ease of use, and the lack of reasons to make their use undesirable from a clinical point of view, the panel was in favor of offering those drugs within the other available options which are usually considered when choosing a migraine preventive treatment. There are no reasons on clinical grounds to postpone the initiation of this treatment. However, first line treatment option should be carefully chosen by physicians considering the patient’s history, comorbidities, and burden of the disease. Headache experts must be able to choose, after discussion with the patient, the therapy that is most appropriate. Comorbid depression and migraine may make preferrable the choice of an antidepressant, comorbid uncontrolled hypertension may favor a beta-blocker or renin angiotensin system inhibitors. Postponing the initiation of CGRP-mAbs, being forced to use strategies which cannot be considered ideal in a patient is a suboptimal treatment paradigm, which does not lead to immediate advantages to individuals with migraine and may favor disease progression and chronicity.

**Table:** 

In individuals with migraine who require preventive treatment, we suggest monoclonal antibodies targeting the CGRP pathway to be included as a first line treatment option.	

#### Expert consensus statement – question 2

**Table:** 

How should other preventive treatments be managed when using monoclonal antibodies targeting the CGRP pathway in individuals with migraine?	

##### Clinical guidance

We have scarce information on how to manage other oral preventive treatments in association with CGRP-mAbs in individuals with migraine. Individuals with migraine who are considered for starting a CGRP-mAb may already be taking other preventive drugs. In this case there is the option to stop the ongoing preventative when starting a CGRP-mAb or to continue the oral preventatives and decide later whether to stop. Benefits and risks of the two options should be considered and discussed with individuals with migraine. Polytherapy can also be considered at a later stage in individuals with migraine who still have a relevant residual migraine burden despite having a clinically meaningful relief with a CGRP-mAb. So far, there is no robust evidence either to support or discard the combination of different migraine preventatives. Withdrawal of other preventive drugs can be done early or later in individuals with migraine showing a favorable clinical response after starting the CGRP-mAb. While as general concept monotherapy is preferrable, some individuals with migraine do not have adequate pain relief with a single drug. In those cases, a combination of different drugs might be considered referring to the previous pharmacological history and comorbidities. Combined treatment might be particularly suitable for patients achieving a substantial relative response (e.g. 50% reduction in monthly migraine days) with CGRP-mAbs with a relevant number of residual migraine or headache days [39]. Due to these considerations, the panel decided not to make an explicit statement either in favor or against combination therapy. and to leave this option to individual considerations.

**Table:** 

In individuals with episodic or chronic migraine there is insufficient evidence to make suggestions regarding the combination of monoclonal antibodies targeting the CGRP with other preventatives to improve migraine clinical outcomes	

#### Expert consensus statement – question 3

**Table:** 

When should treatment efficacy in patients on treatment with anti-CGRP monoclonal antibodies be firstly evaluated?	

##### Clinical guidance

As a rule, treatment can be stopped if it is considered not effective. Available date from RCTs and from observational studies indicated that CGRP-mAbs have a quick onset of action [33, 40–48] as benefits may be evident in some individuals with migraine even in the first days or first week after starting treatment. Data from randomized and real-world studies also showed that there may be an increase in the responder rate over time as a variable proportion of individuals with migraine who do not have an immediate response start to have a favorable response later on with the ongoing treatment [33, 46–50]. The majority of individuals with migraine who can be considered responders can be identified after 3 months [33, 46, 47, 49, 51]. For those reasons we suggest the first evaluation of individuals with migraine to address efficacy to take place after a minimum of three consecutive months of treatment. We recognize that some individuals with migraine may take more time to achieve a relevant benefit. In selected cases decision on treatment maintenance can be readdressed after an additional period of 3 months.

**Table:** 

In individuals with episodic or chronic migraine who start a new treatment with one monoclonal antibody targeting the CGRP pathway we suggest evaluating efficacy after a minimum of 3 consecutive months on treatment	

#### Expert consensus statement – question 4

**Table:** 

When should treatment with anti-CGRP monoclonal antibodies be paused in individuals with migraine?	

##### Clinical guidance

The CGRP-mAbs are challenging the conventional temporal paradigm of migraine prevention. With the conventional oral preventative drugs, individuals with migraine were typically treated for 6 to 12 months in order to minimize side effects and to re-evaluate the underlying disease burden given the intrinsic cyclic course of migraine. Treatments were then repeated over time for a variable duration according to clinical needs. Monthly or quarterly administration of CGRP mAbs is more accepted by individuals with migraine than the daily oral regimen. Moreover, the excellent tolerability profile makes the CGRP-mAbs more suitable for prolonged treatments. So far, there are no studies which provide a clear guidance on the optimal duration of migraine preventive treatments. It is highly probable that a broadly generalizable approach does not exist and that also treatment duration needs to be adapted on a case-by-case strategy or considering homogeneous groups of individuals with migraine. One question is still open as to whether a longer duration of treatment may have a disease-modifying effect in individuals with a long history of chronic migraine and be able to provide a stable reduction of migraine or headache days, even after stopping the treatment.

**Table:** 

In individuals with episodic or chronic migraine we suggest considering a pause in the treatment with monoclonal antibodies targeting the CGRP pathway after 12-18 months of continuous treatment. If deemed necessary, treatment should be continued as long as needed. In individuals with migraine who pause treatment, we suggest restarting the treatment if migraine worsens after treatment withdrawal.	

#### Expert consensus statement – question 5

**Table:** 

Should individuals with migraine and medication overuse be offered treatment with monoclonal antibodies targeting the CGRP pathway?	

##### Clinical guidance

All the available RCTs on chronic migraine included individuals with migraine and medication overuse [13, 17, 20, 22, 23]. CGRP-mAbs were started without specific strategies in the population of individuals with migraine and medication overuse. In those studies, the efficacy of all four mAbs seemed to be independent of whether the patient had medication overuse [52–54]. We have, at this moment, no evidence to indicate that the effect of CGRP mAbs will be different, if preceded or not by detoxification. In clinical practice, some adopt withdrawal strategies before offering preventive medications to individuals with medication overuse and there is some evidence indicating that detoxification is feasible and effective [55]. However, detoxification is not easy and feasible in all individuals with migraine and dedicated resources are needed.

In addition to evidence from RCTs, there is also evidence from real-world studies suggesting that CGRP-mAbs are highly effective even in the absence of prior detoxification in individuals with medication overuse [31, 56] and that the response to CGRP-mAbs does not depend on detoxification [57, 58].

In individuals with migraine and medication overuse there is need of well-designed clinical trials to evaluate the effect of treatment CGRP-mAbs before and after withdrawal of acute medication. Additionally, it should be clarified whether individuals with migraine and medication overuse who do not respond to CGRP-mAbs may benefit from detoxification strategies before initiation of CGRP-mAbs and whether detoxification may change the responder status.

**Table:** 

In individuals with migraine and medication overuse, we suggest offering monoclonal antibodies targeting the CGRP pathway.	

#### Expert consensus statement – question 6

**Table:** 

In individuals with migraine who are non-responders to one monoclonal antibody targeting the CGRP pathway, is switching to a different antibody an option?	

##### Clinical guidance

All the CGRP-mAbs have an excellent tolerability profile. Nevertheless, tolerability issues may appear and make stopping of one CGRP-mAb necessary. If the reported side effect is specific for a given CGRP-mAb (e.g. constipation related to erenumab) [59], switching to a different CGRP-mAb may be appropriate based on clinical experience. Much more complex is the issue of a CGRP-mAb switch for efficacy reasons. Indeed, there is a non-negligible proportion of individuals with migraine who do not have a clinical response after maintaining the treatment for an adequate period [39, 60]. In those individuals with migraine, a switch to a different CGRP-mAb may represent an option. Considerations to support the switch from one CGRP-mAb to another, include differences in the mechanism of action (action on the ligand or on the receptor), difference in administration schedule (monthly versus quarterly) and to a lesser extent difference in formulations (subcutaneous versus intravenous) Eptinezumab is the only CGRP mAb available in an intravenous formulation. From a pharmacological perspective, eptinezumab only requires hours (theoretically even only minutes, given its intravenous administration) to reach its maximum serum concentrations, which is as fast as the gepants, but considerably faster than the other antibodies, which require up to 1 week to reach their maximum levels [61]. So far, there are no RCTs which addressed whether switching between different CGRP-mAbs may offer benefits to non-responder individuals with migraine. Some observational studies provide information to support this possibility [62–64]; however, bias cannot be excluded, and those data cannot be considered sufficient to recommend a switch. We also have to consider that many individuals with migraine, who are non-responders to CGRP-mAbs, have already failed all the other treatment options and so the switch to a different CGRP-mAb may represent the only viable strategy. It is worth to know that in the migraine treatment setting, switch to other drugs in the same class is an accepted strategy for some classes (e.g. switch to one triptan to a different triptan).

Considering the above reported reasons, the panel expressed a consensus statement to recognize the lack of adequate scientific evidence but at the same time we acknowledge that, for some individuals with migraine, a switch may represent the best therapeutic option. RCTs to test a CGRP-mAb switch in individuals with inadequate response to the first CGRP-mAb are needed to provide information on this issue.

**Table:** 

In individuals with migraine with inadequate response to one monoclonal antibody targeting the CGRP pathway, there is insufficient evidence on the potential benefits of antibody switch but switching may be an option.	

#### Expert consensus statement – question 7

**Table:** 

In which individuals with migraine is caution suggested when considering treatment with monoclonal antibodies targeting the CGRP pathway?	

##### Clinical guidance

CGRP-mAbs are unlikely to produce drug interactions which may be particularly relevant in individuals with migraine with comorbidities. Pregnant and nursing women were excluded from RCTs and there is no robust information on the risk for the fetus or the newborn driven by CGRP-mAbs. The limited real-life data available so far have not shown major concerns with the accidental and short-lived exposure to erenumab, galcanezumab, and fremanezumab in pregnancy and lactation [65]. However, caution is needed because experimental data indicate that erenumab crosses the placenta [66]. Moreover, CGRP has an important role in the regulation of uteroplacental circulation; its levels are increased during physiological pregnancy and decreased in pre-eclampsia [67]. Concerns in the use of those drugs in women of childbearing potential are related also to the long (around 1 month) half-life of the CGRP-mAbs that implies that these drugs can only be considered as eliminated from the circulation 6 months after stopping [61]. Information about the potential risk related to an unplanned pregnancy are to be discussed with female individuals with migraine of childbearing potential.

Concerns regarding vascular safety of these drugs were raised considering that CGRP is among the most potent vasodilators in animals and humans and that CGRP-mediated vasodilation is a rescue mechanism in brain as well as cardiac ischemia [68–70]. Additionally, there is experimental evidence that blockade of the CGRP pathway by a small molecule CGRP antagonist may worsen an ischemic stroke [71]. Although, one study did not show an increased risk after the administration of erenumab in individuals with migraine and stable angina [72], data should be taken with caution because of methodological issues [73]. Results from RCTs have not shown potential risks even in longer follow-up; however, it should be considered that patients considered at high vascular risk were generally excluded [74]. So far in real-world studies, no reliable evidence of an association between CGRP-mAbs and vascular events has emerged; but again, in those studies most of the patients were at low vascular risk. Retrospective analysis of postmarketing (spontaneous) case reports of erenumab-associated adverse events, indicated an association between erenumab use and high blood pressure [75] which has led to change in the label for this drug. Given those premises, a case-by-case evaluation is needed when considering the use of CGRP-mAbs in individuals with migraine considered at high vascular risk of with overt history of vascular events. The Expert panel also decided to suggest caution in the use in individuals with migraine with a history of Raynaud phenomenon as some reports have linked the use of CGRP-mAbs to this phenomenon [76–78].

Constipation could be related to CGRP-mAb use due to potential inhibition of gastrointestinal motility, which is regulated by CGRP [79, 80]. Constipation emerged as a frequent adverse event of treatment with galcanezumab and mostly with erenumab, as reported in real-world studies [33, 46, 47]; however, the vast majority of cases was mild and did not lead to treatment stopping. There is a single reported case of paralytic ileus after abdominal surgery in a patient treated with erenumab and with a history of constipation [81]. In the absence of further safety data, caution might be needed when using erenumab in patients with a history of constipation.

**Table:** 

We suggest avoiding monoclonal antibodies targeting the CGRP pathway in pregnant or nursing women. We suggest caution and decision on a case-by-case basis in the presence of vascular disease or risk factors and Raynaud phenomenon. We suggest caution in erenumab use in individuals with migraine and history of severe constipation.	

## Conclusions

The available data confirm that monoclonal antibodies targeting the CGRP pathway appear to be effective and safe for migraine prevention even in the long term. Objective biomarkers of treatment response are still lacking; nevertheless, the available RCTs and real-world data can provide insights on treatment individualization, including treatment duration, combination with other treatments, and the management of safety issues.

## Supplementary Information

**Additional file 1.** Conflicts of interest disclosures.

## Data Availability

There are no original data.

## Abbreviations

CGRP: Calcitonin gene-related peptide
CM: Chronic migraine
EHF: European Headache Federation
EM: Episodic migraine
GRADE: Grading of Recommendation Assessment, Development, and Evaluation
mAb(s): Monoclonal antibody(ies)
RCT(s): Randomized controlled trial(s)
