# Supplementary material for: European Headache Federation guideline on the use of monoclonal antibodies targeting the calcitonin gene related peptide pathway for migraine prevention – 2022 update
Source: J Headache Pain. 2022 Jun 11;23(1):67. doi: 10.1186/s10194-022-01431-x (PMC9188162; doi:10.1186/s10194-022-01431-x)
Supplement: Supplementary file 1 — Additional file 1. Conflicts of interest disclosures. [file 10194_2022_1431_MOESM1_ESM.docx]

Conflicts of interest disclosures

| **Name** | **Employment in Pharma industry** | **Personal fees (fees as advisor or speaker, consultancy, any other)** | **Institutional fees (research support, any other)** | **Stock Option in Pharma Industry** | **Intellectual (participation in study design or analyses of one of the RCTs included in this guideline)** |
| --- | --- | --- | --- | --- | --- |
| Simona Sacco | No | Allergan-AbbVie, Abbott, AstraZeneca, Lilly, Lundbeck, Novartis, Novo-Nordisk, Teva | Novartis, Uriach | No | No |
| Faisal Mohammad Amin | No | Lundbeck, Novartis, Teva and Eli Lilly | No | No | No |
| Messoud Ashina | No | Allergan-AbbVie, Amgen, Eli Lilly, Lundbeck, Novartis, Teva | Lundbeck Foundation, Novo Nordisk Foundation, Novartis | No | Principal investigator for AbbVie, Amgen, Eli Lilly, Lundbeck, Novartis, Teva clinical trials |
| Lars Bendtsen | No | Allergan-AbbVie, Eli Lilly, Lundbeck, Novartis, Teva | Principal investigator for an investigator initiated clinical trial supported by Novartis not related to migraine | No | No |
| Christina Deligianni | No | No | No | No | No |
| Raquel Gil-Gouveia | No | Allergan-Abbvie, Lilly, Lunbeck, Novartis, Teva | Novartis, Amgen, Lundbeck, Bayer, | No | No |
| Zaza Katsarava | No | Allergan-Abbvie, Lilly, Lundbeck, Merck, Novartis, TEVA | Novartis | No | No |
| Antoinette MaassenVanDenBrink | No | Allergan-Abbvie, Lilly, Novartis, Teva | Novartis, Satsuma | No | No |
| Paolo Martelletti | No | Springer, SpringerNature | Novartis | No | No |
| Dimos-Dimitrios Mitsikostas | No | Allergan, Amgen, Bayer, Biogen, Cefaly, Genesis Pharma, GlaxoSmithKline, ElectroCore, Eli Lilly, Merck-Serono, Merz, Mylan, Novartis, Roche, Sanofi- Genzyme, Specifar and Teva | Biogen, Genesis Pharma | No | Princinpal investigator in trials sponsored by Amgen, Eli-Lily, Lundbeck, Novartis, Teva |
| Raffaele Ornello | No | Eli Lilly, Novartis, Teva | Allergan-AbbVie, Novartis | No | No |
| Uwe Reuter | No | Amgen, Allergan-AbbVie, Alder, Lilly, Lundbeck, Novartis, Pfizer, Teva | BMBF, Novartis Pharma (Cherub 01); Amgen, Allergan-AbbVie, Alder, Lilly, Lundbeck, Pfizer, Teva | No | Principal investigator for trials sponsired by Eli Lilly & Novartis |
| Margarita Sanchez- del- Rio | No | Allergan-AbbVie, Lilly, Novartis, TEVA | No | No | No |
| Alexandra J. Sinclair | Director and Chief Scientific officer for Invex Therapeutics (a University of Birmingham start-up company running a trial in Idiopathic Intracranial Hypertension) | Allergan-AbbVie, Amgen, Chiesi, Novartis, Lundbeck | No | Invex Therapeutics (a University of Birmingham start-up company running a trial in Idiopathic Intracranial Hypertension) | No |
| Gisela Terwindt | No | Allerfgan/Abbvie, Novartis, Lilly, Teva, Lundbeck | Not of importance for this review.  Independent support from Dutch Research Council and Dutch Brain and Heart foundation for other work | No | No |
| Derya Uluduz | No | Allergan-AbbVie, Eli Lilly, Novartis | No | No | Investigator in trials by Amgen, Novartis |
| Jan Versijpt | - No | Allergan-AbbVie, Lundbeck, Novartis, Teva | - | - | - |
| Christian Lampl | No | Allergan-Abbvie, Lilly, Lundbeck, Merck, Novartis, Pfizer, TEVA | No | No | No |
